# Targeted Restoration of the Intestinal Microbiota with a Simple, Defined Bacteriotherapy Resolves Relapsing *Clostridium difficile* Disease in Mice

**DOI:** 10.1371/journal.ppat.1002995

**Published:** 2012-10-25

**Authors:** Trevor D. Lawley, Simon Clare, Alan W. Walker, Mark D. Stares, Thomas R. Connor, Claire Raisen, David Goulding, Roland Rad, Fernanda Schreiber, Cordelia Brandt, Laura J. Deakin, Derek J. Pickard, Sylvia H. Duncan, Harry J. Flint, Taane G. Clark, Julian Parkhill, Gordon Dougan

**Affiliations:** 1 Wellcome Trust Sanger Institute, Hinxton, United Kingdom; 2 Rowett Institute of Nutrition and Health, Aberdeen, United Kingdom; 3 London School of Hygiene and Tropical Medicine, London, United Kingdom; Harvard Medical School, United States of America

## Abstract

Relapsing *C. difficile* disease in humans is linked to a pathological imbalance within the intestinal microbiota, termed dysbiosis, which remains poorly understood. We show that mice infected with epidemic *C. difficile* (genotype 027/BI) develop highly contagious, chronic intestinal disease and persistent dysbiosis characterized by a distinct, simplified microbiota containing opportunistic pathogens and altered metabolite production. Chronic *C. difficile* 027/BI infection was refractory to vancomycin treatment leading to relapsing disease. In contrast, treatment of *C. difficile* 027/BI infected mice with feces from healthy mice rapidly restored a diverse, healthy microbiota and resolved *C. difficile* disease and contagiousness. We used this model to identify a simple mixture of six phylogenetically diverse intestinal bacteria, including novel species, which can re-establish a health-associated microbiota and clear *C. difficile* 027/BI infection from mice. Thus, targeting a dysbiotic microbiota with a defined mixture of phylogenetically diverse bacteria can trigger major shifts in the microbial community structure that displaces *C. difficile* and, as a result, resolves disease and contagiousness. Further, we demonstrate a rational approach to harness the therapeutic potential of health-associated microbial communities to treat *C. difficile* disease and potentially other forms of intestinal dysbiosis.

## Author Summary

Pathological imbalances within the intestinal microbiota, termed dysbiosis, are often associated with chronic *Clostridium difficile* infections in humans. We show that infection of mice with the healthcare pathogen *C. difficile* leads to persistent intestinal dysbiosis that is associated with chronic disease and a highly contagious state. Using this model we rationally designed a simple mixture of phylogenetically diverse intestinal bacteria that can disrupt intestinal dysbiosis and as a result resolve disease and contagiousness. Our results validate the microbiota as a viable therapeutic target and open the way to rationally design bacteriotherapy to treat chronic *C. difficile* infections and potentially other forms of persistent dysbiosis.

## Introduction

*Clostridium difficile* is an anaerobic, Gram-positive bacterium that is the major cause of antibiotic-associated diarrhea and a significant healthcare-associated pathogen [1]. *C. difficile* challenges hospital infection control measures by exploiting an infection cycle involving the excretion of highly transmissible and resistant spores that act as an environmental transmission reservoir [2]–[4]. Antibiotic treatment of hospitalized patients is the major risk factor for *C. difficile* colonization and disease that are characterized by a toxin-mediated neutrophil response [5] and a spectrum of outcomes from asymptomatic carriage, severe diarrhea, fulminant pseudomembranous colitis, toxic megacolon and occasionally death [6]. First line treatments for *C. difficile* disease are vancomycin or metronidazole, although in 20–35% of these cases a recurrent disease (relapse or re-infection) follows cessation of antibiotic therapy [7]. More recently, a narrow-spectrum antibiotic, Fidaxomicin, has been shown to cause less damage to the microbiota and lower rates of recurrence compared to vancomycin [8], [9]. This has led to the proposal that *C. difficile* disease is linked to a general imbalance of the intestinal microbiota, often referred to as dysbiosis [10], [11]. Alternatively, probiotic-based approaches that restore intestinal homeostasis are viewed as promising therapies for recurrent *C. difficile* infection [12], [13].

During the past decade distinct genetic variants of *C. difficile* have emerged that are responsible for epidemics within North America and Europe and continue to disseminate globally [14], [15]. Most notable is the “epidemic” variant, genotypically referred to as PCR-ribotype 027 or REA group BI, which is associated with high-level toxin production [16](Figure S1), high rates of recurrence and mortality, and severe hospital outbreaks [17]–[19]. We have recently used whole genome sequencing to demonstrate that isolates within the epidemic *C. difficile* 027/BI clade are genetically distinct from other human virulent *C. difficile*, such as the 017/CF and 012/R variants that are endemic in many hospitals throughout Europe [20], and have likely emerged and spread globally within the past decade [21]–[23]. Although the epidemic *C. difficile* 027/BI variant is now the most common type causing disease in many parts of the world [24] it is not known how this particular variant transmits so effectively and outcompetes other *C. difficile* disease-causing variants [25].

The recent description of *C. difficile* murine infection models that mimic many aspects of asymptomatic carriage, disease and spore-mediated transmission in humans [26], [27] has facilitated experimental investigations into the molecular basis of *C. difficile* disease [28]–[31] and transmission [32]–[34]. Here we use a murine infection model to demonstrate that animals infected via natural transmission with epidemic *C. difficile* 027/BI, but not other human virulent *C. difficile* variants, develop chronic infection and a highly contagious state that persists for months. Persistent infection is linked to intestinal dysbiosis that can be resolved by restoring a diverse intestinal microbiota with bacteriotherapy using a defined, simplified mixture of intestinal bacteria.

## Results

### Epidemic *C. difficile* 027/BI causes chronic, contagious disease in mice

**Figure 1:**
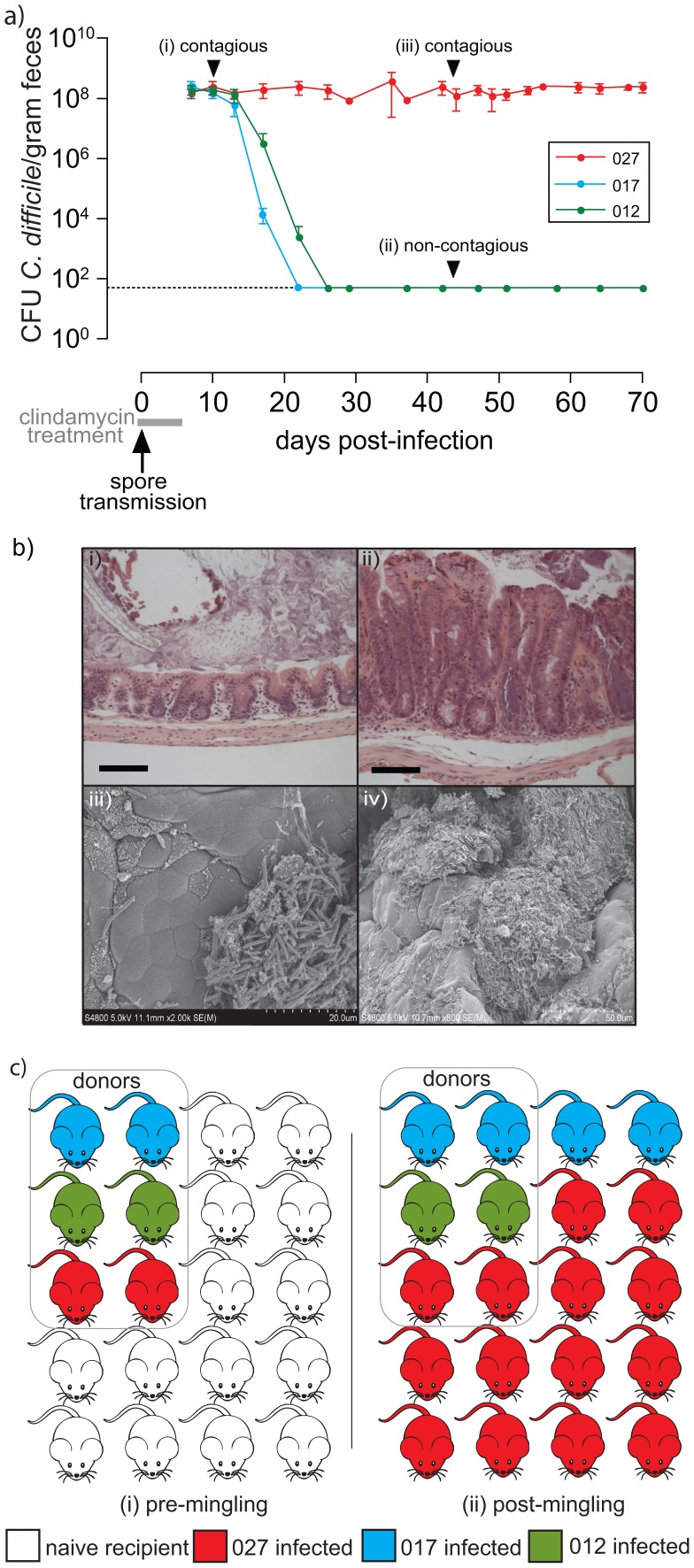
Epidemic *C. difficile* 027/BI causes persistent infection with enhanced transmissibility compared to other virulent variants. a) Representative fecal shedding patterns from C57BL/6 mice (n = 5 mice per group) simultaneously treated with clindamycin and exposed to human virulent *C. difficile* spores to mimic natural transmission. Mice were infected with *C. difficile* ribotype 027 (strain BI-7; n = 300), 017 (strain M68; n = 240) and 012 (strain 630; n = 50). Mice supershedding high-levels of *C. difficile* (>10^8^ CFU/gram fresh feces) are highly contagious (i and iii) whereas mice shedding low-levels of *C. difficile* (ii; <10^2^ CFU/gram fresh feces) are non-contagious (Figure S3). Broken horizontal line indicates culture detection limit of 50 CFU/gram feces. b) i–ii) hematoxylin and eosin staining to compare cecal pathology of i) healthy, clindamycin treated mice to ii) persistent *C. difficile* 027/BI-7 supershedders (day 49 post-infection; C57BL/6) that display signs of hyperplasia, edema and immune cell filtrate. Scale bars represent 100 µm. iii–iv) Scanning electron micrographs of illustrating the presence of *C. difficile* microcolonies (iii) and biofilm-like structures (iv) on the intestinal mucosal surface of persistent supershedders. Scale bars shown in bottom right corner. c) *C. difficile* 027/BI-7 outcompetes *C. difficile* 012/R and 017/CF within susceptible host populations. Shown is the summary of two independent experiments that included 6 *C. difficile* infected donor mice (2 donors infected individually with either *C. difficile* ribotype 012, 017 or 027) housed with 14 naïve recipient mice for 30 days. The transmission rate of *C. difficile* 027/BI-7 infected mice is significantly higher (p<1.1e−4) than that of *C. difficile* 012/630 (p<0.02) or 017/M68 (p<0.22) infected mice.

We infected groups of healthy C57BL/6 mice separately with *C. difficile* PCR ribotypes 012 (strain 630 [23]), 017 (strain M68 [21]) or 027 (strain BI-7 [21]) via spore-mediated transmission and subsequently treated the infected groups with a clinically relevant dose of clindamycin for 7 days. Each of these *C. difficile* variants was isolated from hospital patients with *C. difficile* disease and is resistant to high levels of clindamycin (MIC of >256 mg/L). This mode of infection mimics natural transmission and reproducibly results in high-level excretion of *C. difficile* (>10^8^ CFU/gram feces) (Figure 1a (i)). Mice that shed *C. difficile* at this level are highly contagious (Figure 1a (i)); Figure S2), which we refer to as “supershedders” [35], and must be housed under stringent conditions to contain spore-mediated transmission [27], [33].

Mice infected with *C. difficile* 017/M68 and 012/630 reproducibly (100% for 012, n = 50; >97% for 017, n = 240) lost the supershedder state by 10–14 days post-clindamycin treatment leading to a non-contagious carrier state (<10^2^ CFU/gram feces)(Figure 1a; Figure S2) and resolution of intestinal pathology [27]. In contrast, the majority (>70%, n = 300) of mice infected with epidemic *C. difficile* 027/BI-7 remained as persistent supershedders for months, even in the absence of continued clindamycin treatment (Figure 1a). Persistent supershedders of *C. difficile* 027/BI-7 displayed significant signs of chronic intestinal disease (Figure 1b i–ii) and harbored microcolonies and biofilm-like structures containing *C. difficile* on the intestinal mucosal surface (Figure 1b iii–iv). The cecal tissue of mice infected with epidemic *C. difficile* 027/BI-7 also exhibited a significant up-regulation of pro-inflammatory genes, particularly those known to promote neutrophil infiltration (Figure S3 and Table S1), similar to the human immune response [5].

Thus, epidemic *C. difficile* 027/BI-7 induces a persistent supershedder state, characterized by intestinal disease and a prolonged contagious period in mice, whereas infection with other disease causing variants results in a self-limiting infection leading to a non-contagious carrier state.

### Enhanced transmissibility of epidemic *C. difficile* 027/BI

We hypothesized that since persistent supershedders excrete epidemic *C. difficile* into the environment for a prolonged period compared to other *C. difficile* infected mice, this would increase the transmission of epidemic *C. difficile* in a population of susceptible hosts. To test this hypothesis we housed mice supershedding *C. difficile* 027/BI-7, 017/M68 or 012/630 together with naïve mice for 30 days and then determined the proportion of mice infected with each *C. difficile* variant. After exposure to supershedders, all naïve mice became colonized by *C. difficile* (Figure 1c). Significantly, the majority of naïve recipient mice (12/14) were infected with the epidemic *C. difficile* 027/BI-7 and a minority (2/14) were colonized by *C. difficile* 017/M68 whereas the donor mice remained infected with only the original infecting strain (Figure 1c). Therefore, the ability of epidemic *C. difficile* 027/BI-7 to induce a persistent supershedder state within hosts provides this variant with a competitive advantage over other variants within a susceptible host population.

### Epidemic *C. difficile* 027/BI induces intestinal dysbiosis

Recurrent *C. difficile* disease in humans is associated with a general reduction in intestinal bacterial diversity [10]. We therefore hypothesized that the persistent supershedder state in mice caused by *C. difficile* 027/BI-7 is linked to alterations in the structure of the co-inhabiting bacterial community. To address this hypothesis we analyzed the composition of the intestinal microbiota from mice using 16S rRNA gene sequence profiling of bacterial DNA isolated from fresh fecal pellets.

**Figure 2:**
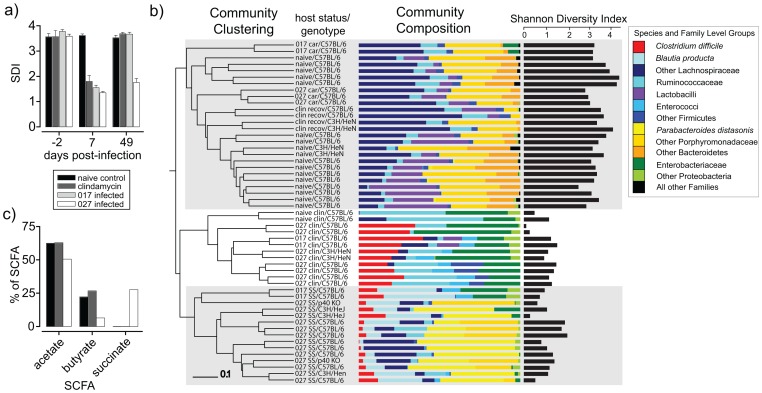
Epidemic *C. difficile* 027/BI-7 induces intestinal dysbiosis in mice. a) Temporal changes in the Shannon Diversity Indices (SDI) of the intestinal microbiota from naïve C57BL/6 mice, clindamycin treated (7 days) naïve C57BL/6 mice or clindamycin treated C57BL6 mice infected with *C. difficile* 027/BI-7 or 017/M68 (n = 2 mice/group). Fecal samples were collected for DNA extraction two days before clindamycin treatment/infection, 7 days post-treatment/post-infection and 49 days post-treatment/post-infection. b) Analysis of 16S rRNA gene sequences (variable regions 2–5) derived from fecal pellets of naïve mice (n = 17), *C. difficile* carriers (n = 5; 35–49 days post-infection), mice undergoing clindamycin treatment (n = 12), mice recovered from clindamycin treatment (n = 4; 42 days after cessation of treatment) and persisting supershedders of *C. difficile* 027/BI-7 (n = 15; 35–49 days post-infection). SS, supershedder; car, carrier; clin recov, mice treated with clindamycin for 7 days and then sampled 42 days later; naïve clin, naïve mice treated with clindamycin for 7 days and then sampled; 027 clin (017 clin), mice infected with *C. difficile* 027/BI-7 (017/M68) and treated with clindamycin for 7 days and then sampled. Community diversity patterns were determined using the Bray Curtis calculator on 336 OTUs (12,316 clones) sharing 98% identity and the Shannon Diversity Index calculated as described. Various murine genetic backgrounds were tested including, C57BL/6, C57BL/6 p40^−/−^, C3H/HeN and C3H/HeJ, as indicated. c) Short chain fatty acid (SCFA) profiles of the intestinal microbiota from naïve C57BL/6 mice, clindamycin-treated C57BL/6 mice that had been allowed to recover for 49 days prior to sampling and *C. difficile* 027/BI-7 supershedding C57BL/6 mice (n = 5 mice/group).

First we assessed the global community structure from individual mice over time by determining the Shannon Diversity Index (SDI), which takes into account species richness (number of species) and evenness (distribution of species). As expected, the intestinal microbiota of naïve, untreated mice was characterized as a diverse bacterial community (∼60 phylotypes/250 clones/mouse), free of *C. difficile*, that was very stable over 50 days and dominated by anaerobic species from the *Bacteroidetes* and *Firmicutes* phyla (Figure 2a). Seven days of clindamycin treatment significantly reduced the SDI of both naïve mice and *C. difficile* (027/BI or 017/CF) infected mice and caused an increase in the proportional abundance of facultative anaerobes such as members of the *Enterobacteriaceae* family and enterococci (Figure 2a). Clindamycin treatment reduced the diversity to 9–12 phylotypes/250 clones/mouse, regardless of *C. difficile* infection, and *C. difficile* clones represented 26.7% (±6.8) of the clone library from infected mice (n = 10)(Figure S4). Interestingly, the SDI and phylum-level compositional structure from naïve mice and *C. difficile* 017/M68 infected mice consistently recovered to pre-clindamycin levels and no *C. difficile* clones were detected from infected mice by 49 days post-clindamycin treatment (Figure 2a). In contrast, infection with epidemic *C. difficile* 027/BI-7 altered the recovery pattern of the intestinal microbiota and instead the species diversity remained very low (10–12 phylotypes/250 clones; n = 15 mice) and *C. difficile* represented 6.8% (±5.2) of the clone libraries at 49 days post-clindamycin treatment (Figure 2a and Figure S4).

We were next interested in defining the supershedder microbiota at the phylotype-level (>98% identity of 16S rRNA gene across variable regions 2–5) so we compared the bacterial communities from mice infected with *C. difficile* 027/BI-7 or 017/M68 as well as the appropriate naïve and clindamycin treatment controls (Figure 2b). Interestingly, the intestinal microbiota from persistent supershedders associated with epidemic *C. difficile* 027/BI-7 infection was consistently simplified in structure (SDI 2.0±0.3 (n = 15) vs. SDI 3.6±0.2 for healthy/naïve mice (n = 17); Figure S4) and, importantly, was distinct in composition from the microbiota of mice undergoing clindamycin treatment, mice that recovered from clindamycin treatment, naïve mice and low-level carriers of 027/BI-7 or 017/M68 (Figure 2b). The emergence of the supershedder microbiota was very robust since it occurred in mice of distinct genetic backgrounds, including C57BL/6, C3H/HeN, C3H/HeJ and in certain C57BL/6 gene knock out mice such as those harboring mutations in the p40 subunit of interleukin 12 (Figure 2b) (Table S2). We noted that the supershedder microbiota consistently contained 16S rRNA gene clones derived from *Blautia producta* and regularly included 16S rRNA gene sequences representative of recognized human opportunistic pathogens, including *Klebsiella pneumoniae*, *Escherichia coli*, *Proteus mirabilis*, *Parabacteroides distasonis* and *Enterococcus faecalis*. We subsequently confirmed the presence of each organism by direct culture and sequence analysis of their 16S rRNA genes (unpublished data). Interestingly, all of these bacterial species have also been identified within the microbiota of humans with *C. difficile* disease using culture dependent [36] and culture independent [10] methods.

Short chain fatty acids (SCFA) are the end products of bacterial fermentation in the intestines and serve as important nutrients for the host [37]. Imbalances in SCFA levels, particularly butyrate and acetate, have been associated with chronic intestinal diseases [38]. To investigate the functional consequence of the simplified intestinal community of persistent supershedders we next profiled the SCFAs present within the large intestine of mice. Interestingly, the microbiota from supershedder mice produced less SFCAs (69.4 µmol/gram cecal contents) compared to that from naïve mice (140.8 µmol/gram) and naïve mice that were given clindamycin and then allowed to recover for 49 days (138.0 µmol/gram). Further, the supershedder microbiota was associated with an altered SCFA profile compared to naïve and clindamycin treated mice that was characterized by a substantial proportional reduction in butyrate and acetate and an increase in succinate levels (Figure 2c), correlating with an increase in the levels of *P. distasonis* (succinate producer) [39]. Thus, we demonstrate that epidemic *C. difficile* 027/BI-7 maintains intestinal dysbiosis in mice after clindamycin treatment that is characterized by a simplified intestinal bacterial community, the presence of opportunistic bacteria and markedly altered SCFA production.

### Fecal transplantation resolves relapsing *C. difficile* 027/BI disease and contagiousness

**Figure 3:**
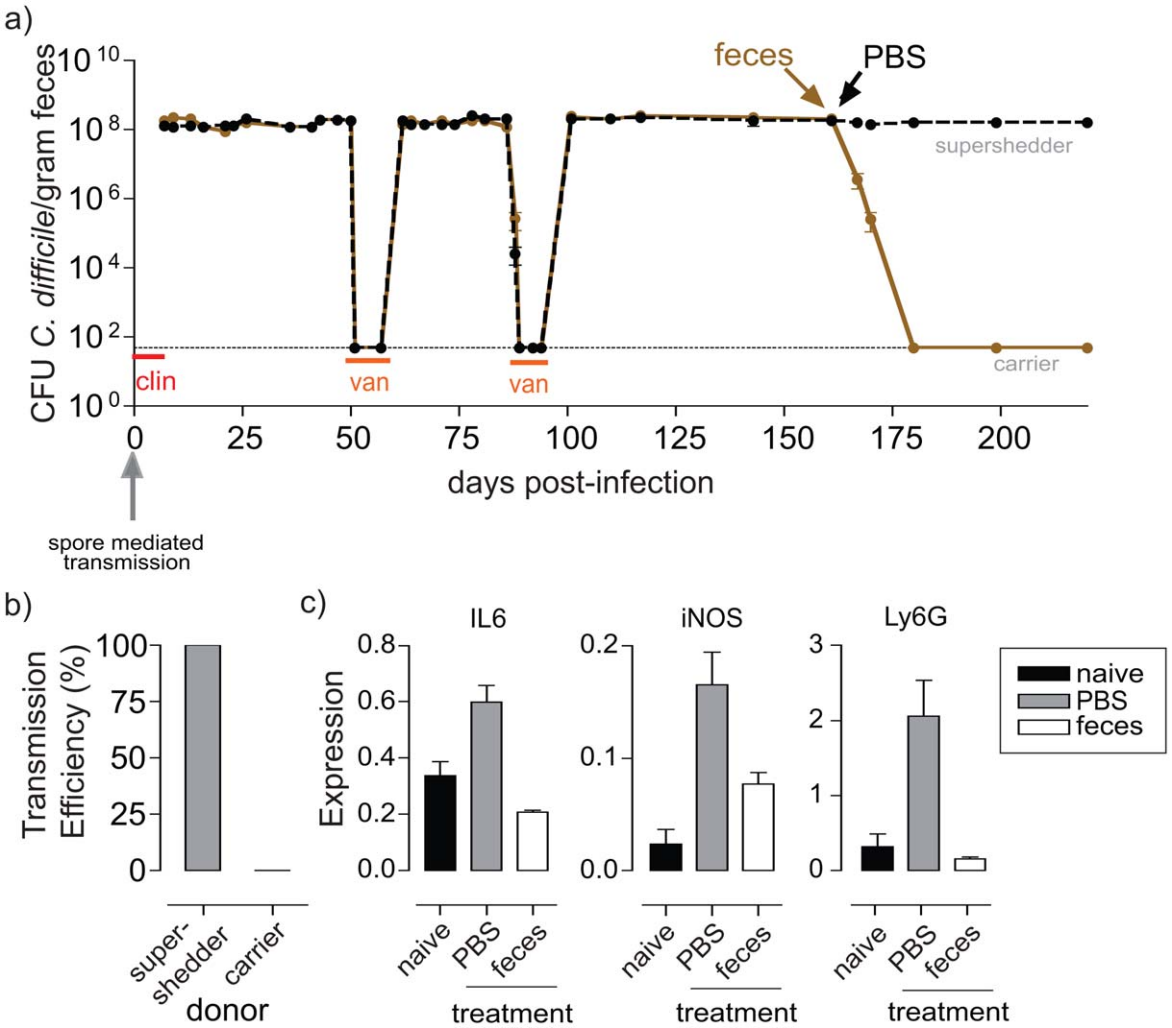
Fecal transplantation resolves relapsing epidemic *C. difficile* 027/BI-7 disease and host contagiousness. a) *C. difficile* shedding patterns from mice (average shedding from 5 mice/cage) demonstrating that epidemic *C. difficile* infection is refractory to vancomycin treatment (van) and results in a relapsing supershedder state. Fecal transplantation suppresses high-level *C. difficile* 027/BI-7 shedding (brown) whereas PBS administration had no impact on *C. difficile* 027/BI-7 shedding levels (black). Toxins were detected in the feces of supershedders but not in the feces of carriers using the ToxA/B Quikchek (Techlab, Blackburg, VA, USA). Broken horizontal line indicates culture detection limit of 50 CFU/gram feces. b) Supershedder mice efficiently transmit *C. difficile* to naive mice whereas mice treated with feces and transformed to carriers become poor donors of infection to naive mice. Transmission efficiency refers to the percentage of naïve recipient mice (n = 10/group) that became infected with *C. difficile* 027/BI-7. c) Quantitative RT-PCR of RNA extracted from supershedder mice cecal tissue showing high-level expression of the proinflammatory genes IL-6, iNOS and Ly6G, which were suppressed to levels comparable to naive mice after fecal transplantation. Cytokine expression was normalized to Gapdh and is shown as relative values.

Next we attempted to clear *C. difficile* 027/BI-7 from persisting supershedders with a 10-day treatment of oral vancomycin. We found that vancomycin treatment of supershedders rapidly suppressed *C. difficile* excretion to below the culture detection limit (Figure 3a), as expected because *C. difficile* 027/BI-7 is susceptible to vancomycin. However, cessation of vancomycin treatment was followed within 5–7 days by a relapse (by the same strain) to high-level *C. difficile* shedding (>10^8^ CFU/gram) in all mice (n = 120)(Figure 3a). Relapse occurred even after mice were aseptically moved to individual sterile cages to reduce host-to-host transmission and re-colonization by environmental spores. Interestingly, the SDI of the intestinal microbiota from relapsed mice remained low (2.1–2.2) and the resident bacteria included opportunistic species (i.e. *E. faecalis*, *E. coli* and *B. producta*)(data not shown).

Fecal transplantation, the administration of homogenized feces from a healthy donor, is a promising alternative therapy for recurrent *C. difficile* disease in humans [40]–[42], so we therefore tested the ability of bacteriotherapy to suppress the *C. difficile* supershedding state. Remarkably, a single treatment via oral gavage of *C. difficile* 027/BI-7 supershedding mice with homogenized feces from a healthy donor rapidly suppressed *C. difficile* shedding levels to below the detection limit within 5–7 days and, in contrast to vancomycin therapy, this lasted for months (Figure 3a). Using this protocol we consistently found that fecal transplantation was highly effective and indeed suppressed the supershedder state in 23 out of 25 attempts.

Suppression of *C. difficile* shedding levels was associated with a significant loss of contagiousness as demonstrated by the inability of treated mice to transmit *C. difficile* to other naïve mice (Figure 3b). Further, fecal transplantation was consistently associated with a resolution of intestinal pathology and a reduction in the expression of proinflammatory genes (Figure 3c). Therefore, intestinal dysbiosis caused by epidemic *C. difficile* is refractory to vancomycin therapy but can be suppressed with feces of a healthy individual leading to resolution of disease and contagiousness.

### Rational design of a simple, defined bacteriotherapy

**Figure 4:**
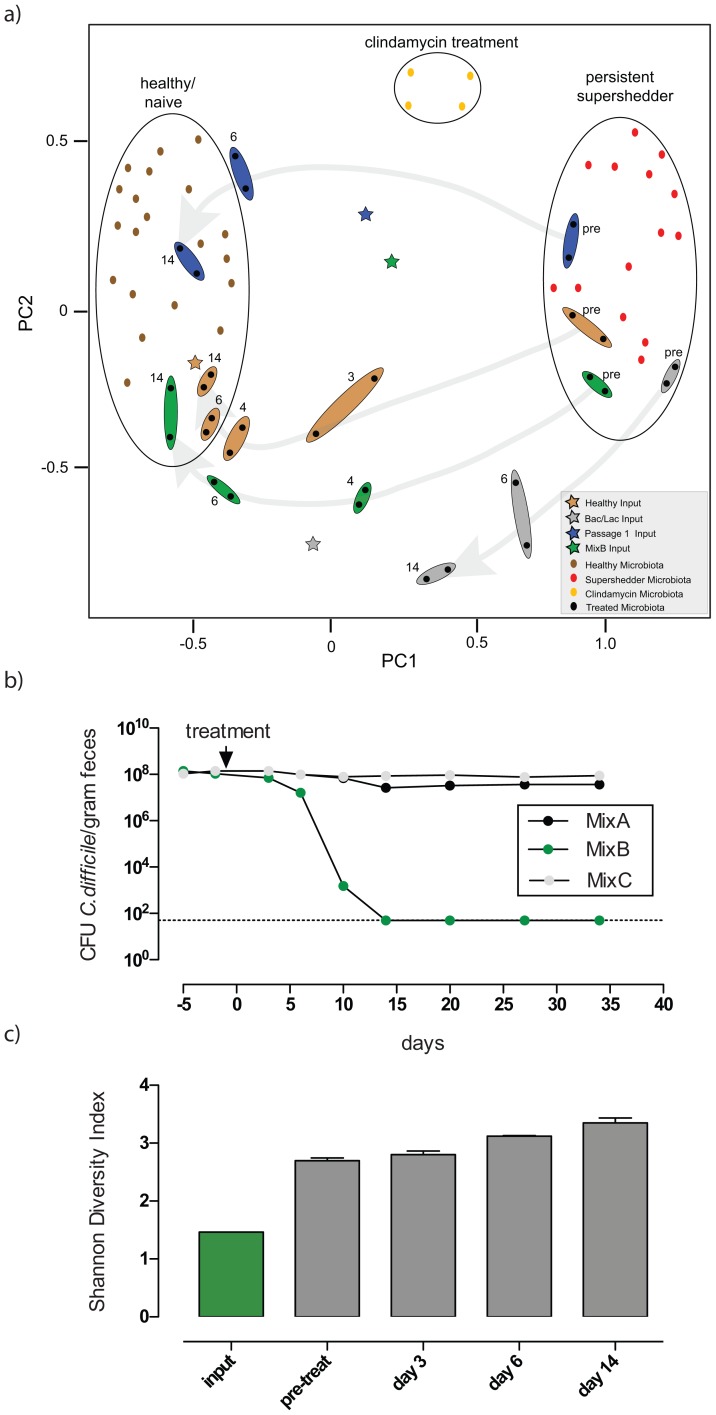
Effective bacteriotherapy re-establishes a healthy, diverse microbiota profile in epidemic *C. difficile* 027/BI supershedder mice. **a**) Principal component analysis of the 16S rRNA gene sequences demonstrates that distinct microbiota profiles (circled) are associated with “healthy/naïve” mice, mice undergoing “clindamycin treatment” and “persisting supershedders” of *C. difficile* 027/BI-7. PC1 and PC2 account for 38% of the variation. Each symbol represents one microbiota (dot) or treatment (star) community. Treatment of supershedder mice with feces from healthy mice, the cultured fecal derivative or mixtures of defined, cultured bacteria are as indicated: brown - shading for healthy feces, blue - shading for fecal derivatives culture passaged once, green - shading for mixture of six suppressive bacteria (MixB) and grey - shading for *Bacteroides/Lactobacillus* mixture. The symbol representing the *Bacteroides/Lactobacillus* treatment is based on culturing counts and modified to reflect the relative abundance of each organism in the mixture. Next to the shading: pre = pre-treatment; 3 = 3 days post-treatment; 4 = 4 days post-treatment; 6 = 6 days post-treatment; 14 = 14 days post-treatment. Grey background arrows indicate the shifts in the microbiota profiles of treated mice over a 14-day period. **b**) Fecal shedding profiles from supershedder mice (n = 5/group) that were treated with MixA, MixB or MixC (Table S3). c) Shannon Diversity Indices of the intestinal microbiota of supershedders pre- and post-treatment (day 3, 6 and 14) with MixB and that of the corresponding input community.

Principal component analysis (PCA) further confirmed that distinct intestinal microbiota profiles are associated with either “healthy/naïve” mice, “persistent supershedders” or mice undergoing “clindamycin treatment” (Figure 4a). Suppression of *C. difficile* shedding levels after fecal transplantation shifted the recipients' microbiota to a composition similar to that of the healthy input bacterial community (Figure 4a; brown shaded dots and star) and this was closely linked to a rapid increase in species diversity (Figure S5). In comparison, treatment of supershedders with PBS, autoclaved feces, fecal filtrate, SCFAs or laboratory *E. coli* had a negligible effect on *C. difficile* shedding levels (Figure S6).

Consequently, we reasoned that there are key bacteria within the microbiota of healthy mice that are responsible for suppressing the *C. difficile* 027/BI supershedder state. To identify candidate bacteria we passaged healthy feces overnight in nutrient broth at 37°C to reduce the community complexity (Figure S7) and to enrich for readily culturable bacteria. Treatment of supershedder mice with cultured fecal derivatives serially passaged twice (Passage 1 and 2) effectively suppressed the supershedder state (Figure S8) and shifted their microbiota composition towards a healthy microbiota profile (Figure 3a). However, a third passage (Passage 3) was dominated by *Enterococcus* spp. and *Enterobacteriaceae* spp. and generally resulted in a loss of the protective effects of the fecal derivative against the *C. diffiicile* 027/BI supershedder state. These results confirm the presence of specific culturable bacteria within the microbiota of healthy mice that can suppress *C. difficile* 027/BI infection as effectively as whole fecal bacteriotherapy.

Next, we cultured a diverse collection of 18 bacterial species from the Passage 1 fecal derivative, including representatives of the four phyla that constitute the majority of the mammalian intestinal microbiota (*Firmicutes*, *Bacteroidetes*, *Actinobacteria* and *Proteobacteria*; Table S3). Since the supershedders' microbiota contained a skewed profile of dominant bacterial phyla (Table S2), we reasoned that inoculation of supershedders with a phylogenetically diverse bacterial mixture could potentially trigger recovery of the intestinal ecosystem and disrupt the stability of the supershedder microbiota. Therefore, we treated supershedders with different combinations of phylogenetically diverse bacterial mixtures (mixtures summarized in Table S3). Many of the combinations failed (see below) but we ultimately identified a mixture of six bacteria that effectively and reproducibly (20/20 mice) suppressed the *C. difficile* 027/BI supershedder state (“MixB”; Figure 4b). Significantly, treatment of supershedders with the MixB bacteria shifted the recipients' intestinal microbiota to the profile of a healthy profile (Figure 4a) and triggered an increase in bacterial diversity (Figure 4c) that was associated with resolution of intestinal disease and contagiousness. Analysis of 16S rRNA gene sequences, and of cultured isolates, derived from treated mice confirmed the presence of five of the six MixB bacteria in the feces during days 6–14 post-treatment (Table S2). Much of the increased diversity, however, was derived from commensal bacteria that were present at low levels pre-treatment (Table S2), suggesting that the MixB bacteria had disrupted colonization by *C. difficile* 027/BI and the other members of the supershedder microbiota by triggering an expansion of the suppressed health-associated bacteria and a re-distribution of the microbiota to a healthy composition.

Significantly, and in contrast to the results with MixB, treatment of mice with further subdivisions of this bacterial mixture, including the MixB bacteria administered individually, or mixtures containing six or seven other cultured bacterial strains had a negligible impact on the supershedder state (Figure 4b). To further illustrate the particular effectiveness of our MixB collection of strains, treatment of supershedders with a *Bacteroides*/*Lactobacillus* mixture, representative of more traditional probiotic bacterial groups [43], [44], failed to resolve the supershedder state and restore the recipients' microbiota to a healthy profile (Figure 4a and Figure S9). Thus, we rationally defined a novel, simple mixture consisting of six phylogenetically diverse intestinal bacterial strains that can resolve *C. difficile* 027/BI infection in mice.

### Dominant supershedder and bacteriotherapy bacteria are phylogenetically distinct

**Figure 5:**
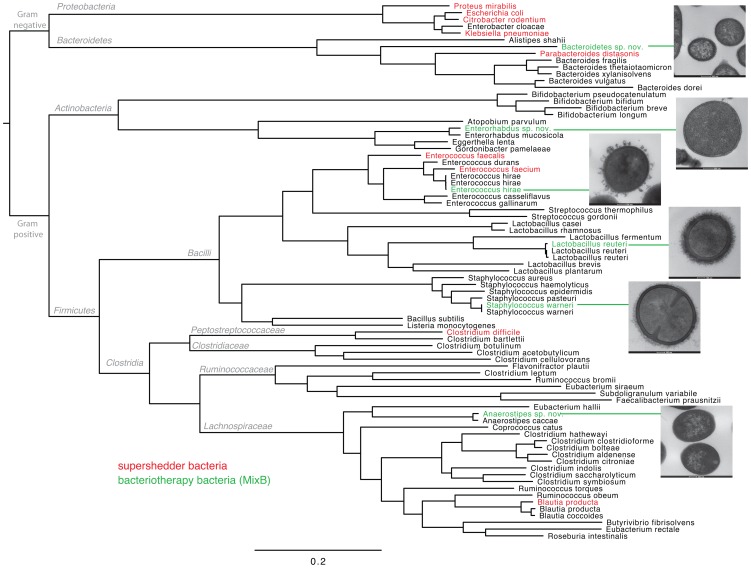
Whole genome (maximum likelihood) phylogeny of intestinal bacteria demonstrating the phylogenetic placement of disease-resolving bacteriotherapy bacteria (MixB) and the dominant members of the supershedder microbiota. Maximum likelihood phylogeny produced using FastTree from the concatenated protein sequence of 44 common genes (See methods). Species names marked in green indicate members of the suppressive MixB mixture, names marked in red indicate species that were commonly detected in the feces of supershedding mice, names in black are reference genomes from common intestinal bacteria that were included to provide phylogenetic context to the tree. Taxonomic designations are given at the relevant branch nodes. Adjacent pictures are transmission electron micrographs of sectioned bacterial strains that constitute MixB. Methods for sample processing and imaging have been described [33]. Scale bars are shown below bacteria.

Next we wanted to fully define the identity of the six bacterial strains present in MixB (Table S3) and to discern their relationship to the dominant members of the supershedder microbiota. To do so we sequenced the genomes of the six MixB bacteria (and their closest equivalent human-derived species) and performed a phylogenetic comparison to the dominant members of the supershedder microbiota and reference intestinal bacterial genomes representative of the mammalian microbiota (Figure 5 and Table S4). Based on this analysis we determined that MixB includes three previously described species, *Staphylococcus warneri*, *Enterococcus hirae*, *Lactobacillus reuteri*, and three novel species, *Anaerostipes* sp. nov., *Bacteroidetes* sp. nov. and *Enterorhabdus* sp. nov. (Table S3). This mix of bacteria is therefore phylogenetically diverse, including both obligate and facultative anaerobic species, and represents three of the four predominant intestinal microbiota phyla. Importantly, these species appear to be common inhabitants of the mouse intestine in health and they are phylogenetically distinct from the dominant members of the supershedder microbiota (Figure 5). Given the demonstrated ineffectiveness of autoclaved feces, fecal filtrates, SFCAs and individual bacterial strains it therefore appears that displacement of *C. difficile* and the supershedder microbiota may require competition from a phylogenetically diverse and physiologically distinct collection of living bacteria.

## Discussion

**Figure 6:**
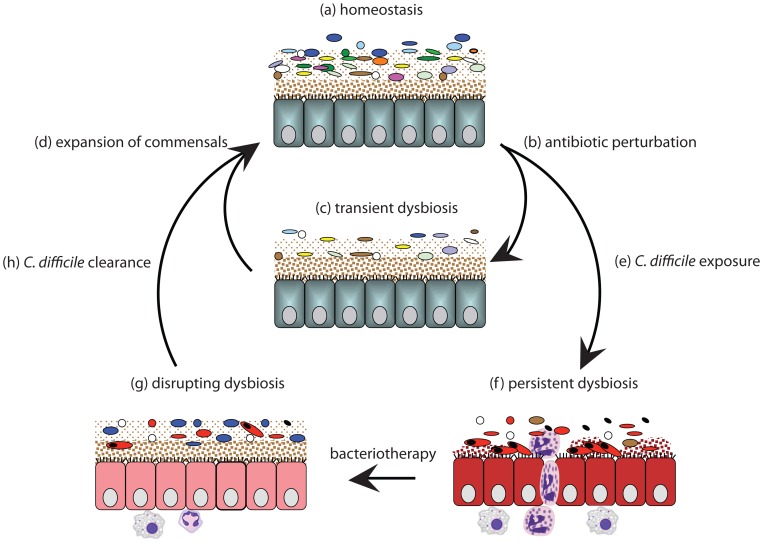
Proposed model for establishment of *C. difficile*-mediated dysbiosis and successful bacteriotherapy. Intestinal homeostasis (a) is characterized by lack of pathology and a diverse, stable microbiota that produces SCFA via fermentation. Antibiotic perturbation (b–c) kills susceptible bacteria resulting in a simplified community structure (and reduced SCFA production) and a loss of colonization resistance. In the absence of opportunistic infection, the microbiota generally rebounds in diversity and SCFA production (d) to re-establish homeostasis and colonization resistance (a). However, exposure to *C. difficile* (e) after antibiotic perturbation (b) can lead to persistent dysbiosis (f) that is characterized by a pathogenic microbial community, reduced SCFA and pathology. Bacteriotherapy disrupts dysbiosis (g) leading to the clearance of *C. difficile* (h) and re-establishment of intestinal homeostasis (a).

We demonstrate that epidemic *C. difficile* 027/BI effectively maintains intestinal dysbiosis after clindamycin treatment, altering the intestinal ecosystem to outcompete health-associated intestinal bacteria. In contrast, neither *C. difficile* 017/CF nor 012/R induced persistent dysbiosis, presumably because they are less virulent in mice. There are large differences between the genomes of the *C. difficile* 027, 012 and 017 lineages [21]–[23] that could account for such differences, including the presence of a binary toxin in the 027/BI lineage [28], that warrant further investigation. As a result, epidemic *C. difficile* 027 is shed into the environment for a greater period compared to other human virulent variants, increasing its likelihood of infecting a susceptible host. This model explains how epidemic variants, like the *C. difficile* 027/BI-7 clade [21], [22], can quickly become the dominant variant within a host population. Below we propose a model to explain the establishment of persistent dysbiosis by epidemic *C. difficile* 027/BI and the successful resolution of *C. difficile* infection by bacteriotherapy (Figure 6).

Antibiotic perturbation of the intestinal microbiota is one of the major risk factors for *C. difficile* colonization and disease [45]. We show that in the absence of *C. difficile* infection clindamycin treatment initially reduces the complexity of the murine microbiota (Figure 6 b and c) before the diversity recovers to a level comparable to the original community by 2–3 weeks post-treatment (Figure 6 d and a) [27]. The majority of studies in humans [46], [47] and mice [27], [48]–[51] have also shown that the diversity of the microbiota is initially diminished by a variety of antibiotic treatments before the microbiota diversity re-establishes. However, there is variability in diversity recovery time and this is likely due to differences in the initial microbiota composition, the host's genetics/immune system status, the spectrum and dose of the antibiotic used [51] and the presence of bacteria in local environment that can potentially re-colonize the host [48].

After antibiotic treatment there is a transient period where colonization resistance is reduced and the host is very susceptible to infection by such pathogens as *C. difficile* [30], [52] or *S.* Typhimurium [50], [51], [53](Figure 6e). The antibiotic-induced susceptible period allows environmental *C. difficile* spores to colonize [32]–[34] or intestinal *C. difficile* to expand from a low-level carrier state [27]. We show that after colonization by environmental spores (Figure 6 e) epidemic *C. difficile* induces a strong neutrophil response compared to other human virulent variants of *C. difficile* (Figure 6f), possibly due to strain differences in toxin production/activity [16], [54] or other genetic differences [21], [22]. The ability of epidemic *C. difficile* 027/BI to induce an inflammatory response promoted the emergence of a distinct microbiota that contained low diversity and recognized human opportunistic pathogens (Figure 6f). The dysbiotic microbiota also produced less SCFAs in tandem with a marked proportional reduction in butyrate and acetate. Butyrate is the major energy source for enterocytes and thereby indirectly supports host response mechanisms [38]. Thus, by limiting the energy resources available to the mucosal epithelium it is possible that the supershedder microbiota may be promoting it's own stability.

Restoration of a healthy microbiota with fecal transplantation is viewed as a promising alternative treatment for recurrent *C. difficile* disease and other forms of intestinal dysbiosis [12], [55], but it is not widely used because of the time required to identify a suitable donor, the risk of introducing opportunistic pathogens as well as a general patient aversion [56]. Thus, the development of a murine model that recapitulates many features of fecal transplantation in humans with recurrent *C. difficile* disease provides a valuable surrogate to understand the basic mechanisms of successful fecal transplantation and also as a basis to develop standardized treatment mixtures (i.e. bacteriotherapy). Using our model we rationally identified a simple mixture of six phylogenetically diverse bacteria that can trigger the expansion of health-associated commensals that appear to be suppressed during persistent dysbiosis (Figure 6 g) and the subsequent displacement of epidemic *C. difficile*, and the supershedder microbiota, to resolve intestinal disease and contagiousness (Figure 6 h and d). Tvede and Rask-Madsen [36] previously demonstrated that a mixture of ten different facultatively aerobic and anaerobic bacteria was able to resolve *C. difficile* infection in a small number of human patients. However, this initial success does not appear to have been maintained and, at present, defined bacteriotherapy mixtures with proven efficacy are sorely lacking.

Our results highlight the malleability and therapeutic potential of health-associated microbial communities and suggest that the administration of a phylogenetically diverse mixture of bacteria is a critical early trigger for the recovery of the suppressed microbiota. Indeed, isolates within “MixB” failed to trigger a response when administered individually, suggesting that diversity is important. Species composition appears to be equally significant since other defined mixtures of strains failed to resolve disease. Crucially however, by identifying the persistent supershedder microbiota, we were able to guide our strain selection by avoiding the use of species that appear to be associated with *C. difficile* during supershedding infection. Following these principles we believe that it is likely that many distinct combinations of bacterial strains will have the potential to treat recalcitrant or recurring *C. difficile* infection. These observations open the way to rationally harness the therapeutic potential of health-associated microbial communities to treat recurrent *C. difficile* disease and transmission in humans, and potentially other forms of disease-associated dysbiosis.

## Materials and Methods

### Ethics statement

All animal procedures were performed in accordance with the United Kingdom Home Office Inspectorate under the Animals (Scientific Procedures) Act 1986. Ethical approval for these procedures were granted by the Wellcome Trust Sanger Institute's Ethical Review Committee.

### Bacterial culturing

*C. difficile* strains BI-7 (genotype 027/BI; clindamycin^R^, thiamphenicol^R^, erythromycin^S^, tetracycline^S^, ciprofloxacin^R^, vancomycin^S^), M68 (genotype 017/CF; clindamycin^R^, thiamphenicol^S^, erythromycin^R^, tetracycline^R^, ciprofloxacin^R^, vancomycin^S^) and 630 (genotype 012/R; clindamycin^R^, thiamphenicol^S^, erythromycin^R^, tetracycline^R^, ciprofloxacin^S^, vancomycin^S^) have been described [21], [27]. The BI-7 culturing of *C. difficile* for infections and from feces was described previously [27]. To isolate the intestinal bacteria from mouse feces or passaged fecal derivatives, the samples were serially diluted in sterile PBS, plated on a panel of nutrient agar plates; Luria Bertani, Brain Heart Infusion, Man Rogosa Sharpe, Fastidious anaerobic media, Columbia base media supplemented with 10% defribrinated horse blood, Wilkins-Chalgren anaerobic media (all media from Becton, Dickinson, Oxford, UK) and grown either aerobically or anaerobically at 37°C for 24–72 hours. Distinct colony types were isolated, culture purified and genomic DNA was isolated to sequence the 16S rRNA gene using broad range primers as described in the microbiota section below. 16S rRNA gene sequences were compared to the GenBank and RDP databases to identify the bacterial species.

### TcdA ELISA

*C. difficile* cultures were grown in Wilson's broth [27] with shaking for 30 h, pelleted by centrifugation and supernatant was removed for TcdA quantification. Microtitre plates (96 well) were coated with capture antibody by adding 50 µl/well of a 2 µg/ml solution of anti-TcdA (TGCBiomics GmbH, Mainz, Germany) in PBS, and incubating overnight at 4°C. Plates were then washed three times in 0.05% Tween20 in PBS (PBS-T) and blocked with 200 µl 1% BSA (bovine serum albumin) in PBS for 2 h at room temperature. Purified TcdA from *C. difficile* strain VPI10463 (TGCBiomics GmbH, Mainz, Germany) was diluted in 1% BSA-PBS (50 µl/well) and used to construct a standard curve. Culture filtrates were diluted as above in order to generate readings within the linear range of the standard curve. Plates were then incubated at room temperature for 2 h, followed by washing in PBS-T as above. The detection antibody (rabbit anti-*Clostridium difficile* toxin A; antibodies-online GmbH, Aachen, Germany) was diluted 1∶5000 in 1% BSA-PBS, added to wells (50 µl/well) and incubated for 2 h at room temperature. After washing, polyclonal swine anti-rabbit IgG conjugated to horseradish peroxidase (Dako, Cambridgeshire, UK) was diluted 1∶1000 in 1% BSA-PBS, added to the wells (50 µl/well) and incubated for 2 h at room temperature. Finally, plates were washed and 100 µl 3,3′,5,5′-tetramethylbenzidine (TMB; Sigma Aldrich, Dorset, UK) substrate was added for 30 min at room temperature in the dark. 50 µl 0.5 M H_2_SO_4_ was added to stop the reaction. Absorbance was then measured at 450 nm on a FLUOStar Omega (BMG Labtech, Bucks, UK).

### Mouse infections

Female mice between 5–9 weeks of age and from the genetic backgrounds C57BL/6, C57BL/6 p40^−/−^, C3H/HeN and C3H/HeJ were routinely used. Mice to be used as *C. difficile* spore donors were infected with 10^5^ *C. difficile* cells via oral gavage and immediately clindamycin (250 mg/L; Apollo Scientific Ltd, Chesire, UK) was added to the drinking water for 1 week to induce high-level spore excretion. To infect experimental mice, one petri dish of contaminated bedding was removed from spore donor cages, placed into recipient mice cages and clindamycin (250 mg/L) was added to the drinking water for 1 week to induce the supershedder phenotype. To infect germ-free C3H/HeN mice, the feces of supershedder mice was collected, diluted in serial PBS and inoculated into mice via oral gavage. To suppress infection, vancomycin (300 mg/L; Sigma Aldrich, York, UK) was added to the drinking water for 10 days. To assess impact of infection, mice were sacrificed at indicated times and cecal tissue was aseptically collected and fixed for pathology as described [27], or fixed for RNA extractions by immersing samples in RNA-later (Applied Biosystems, Warrington, UK).

### Bacteriotherapy treatment

To prepare input for bacteriotherapy, 1 gram of fresh feces was collected from 5 naïve mice, homogenized in 5 ml of sterile PBS and centrifuged for 30 seconds at 14,000 RPM to pellet the particulate matter. The supernatant slurry was collected and 200 µl was gavaged into each mouse within 30 minutes of excretion. To create the defined bacterial mixtures, individual bacteria were grown in Wilkins-Chalgren broth (*Lactobacillus* in Man Rogosa Sharpe broth) for 48–72 hours under anaerobic conditions at 37°C. Bacteria were harvested by centrifugation and re-suspending the pellet in 2 mls of sterile, pre-reduced PBS. Approximately 10^10^ of each bacterium was gavaged into each mouse in a 200 ul volume. To passage healthy feces, two fecal pellets (∼50 mg) were collected aseptically and immediately placed into 20 ml of standing Wilkins-Chalgren Anaerobic broth or Luria broth that was pre-warmed to 37°C under aerobic or anaerobic conditions. Fecal pellets were physically disrupted within the broth using a sterile pipette tip and subsequently incubated standing for 16 hours. For serial passage, 200 ul of the fecal derivative was inoculated into fresh broth and grown as described. For inoculations, the 20 ml cultures were pelleted and then re-suspended into 2 ml of sterile PBS pre-warmed to 37°C under aerobic or anaerobic conditions. Based on visual counts, approximately 4×10^8^ (anaerobic passage) and 8×10^8^ (aerobic passage) bacteria were gavaged into each mouse in a 200 µl volume.

### Microarrays

RNA purification from cecal mucosal tissue was performed using a Qiagen RNeasy mini kit (Qiagen, Austin, TX, USA) according to the manufacturer's protocol. Quality control and quantification were performed using Bioanalyzer 2100 (Agilent Technologies, Palo Alto, CA, USA) and Nanodrop ND100 (Nanodrop Technologies, Wilminton, DE). RNA samples were then amplified and labelled using the Illumina TotalPrep 96 kit (Ambion, Austin, TX, USA) and hybridized onto Illumina Mouse WG-6-V2 Beadchips (Illumina, San Diego, CA, USA). The chips were scanned on an Illumina BeadArray Reader and raw intensities were extracted using Illumina BeadStudio Gene Expression Module.

Normalization and analysis of the microarrays were performed using GeneSpring X software (Agilent Technologies, Berkshire, UK). Normalization procedures utilized were quantile normalization and median of all samples baseline correction. For each comparison, differentially expressed genes were defined as having a fold change ≥2 and a FDR (false discovery rate) corrected p-value≤0.05. Adjusted p-values were calculated using the Benjamini and Hochberg method [57].

### RT-PCR

Quantitative expression analysis was performed by real-time TaqMan RT-PCR on the ABI PRISM 7900HT Sequence Detection System (Applied Biosystems, Warrington, UK) as described previously [58]. Expression of IL-6, iNOS and Ly6G was normalized to Gapdh mRNA. TaqMan primers and probes were designed to span exon junctions or to lie in different exons to prevent amplification of genomic DNA, as described [58]. Primer and probe sequences are shown in Table S3. Probes were labelled with the reporter dye FAM at the 5′- and the quencher dye TAMRA at the 3′-end.

### Transmission experiments

Protocols to test the contagiousness of infected donors (supershedders or carriers) have been described [27]. To compare the contagiousness of different *C. difficile* strains mice infected with either *C. difficile* 012 (strain 630), 017 (strain M68) and 027 (strain BI-7) (immediately after cessation of 7 days of clindamycin treatment) were co-housed with 7 naïve recipient mice for 30 days. Experiments were repeated for a total of 14 naïve mice. To determine if recipient mice were infected with *C. difficile* they were individually placed (aseptically) in sterile cages for 3 days and given clindamycin in their drinking water for 4 days [27]. Afterwards, feces was collected from individual mice and *C. difficile* enumerated by standard methods [27]. Antibiotic resistance profiles were used to determine which *C. difficile* strain had infected mice.

### Analysis of microbiota

Fecal DNA extraction, clone library construction and sequencing were carried out as described previously [27], [59]. Briefly, DNA was extracted from fecal samples using the FastDNA SPIN Kit for Soil and FastPrep machine (MP Biomedicals, Solon, OH) and 16S rRNA genes amplified using primers 7F (5′–AGA GTT TGA TYM TGG CTC AG-3′) and 1510R (5′-ACG GYT ACC TTG TTA CGA CTT-3′). The 16S rRNA genes were then cloned into *E. coli* using pGEM-T Easy Vectors (Promega UK, Southampton, UK) and 284 clones per sample were picked for sequencing (covering regions V2–V5) using an ABI 3730. Sequences were aligned using the RDP aligner [60] and these alignments were manually curated in the ARB package [61] before further analysis. Otherwise, sequences were checked and classified as described previously [62]. In total 19,991 sequences were generated and these were deposited in GenBank (accession numbers JF241944–JF260864 and HE605382–HE608150).

The species diversity in each sample was measured by calculating the Shannon Diversity Index, which takes into account both species richness and relative proportional abundance (evenness), using the mothur software package [63]. Rarefaction curves and Chao1 estimates of total bacterial diversity were also calculated in mothur [63].

Cluster dendrograms and PCA plots were based on a master alignment, which was built using the RDP aligner and subjected to manual curation. Using this alignment a distance matrix, with Felsenstein correction, was created using ARB. The distance matrix was then used as an input for DOTUR [64] using a 98% identity cut-off under the default furthest-neighbor setting. Sequences with >98% phylogenetic similarity were regarded as belonging to the same OTU. These OTUs were then used to calculate cluster dendrograms, using the Bray Curtis calculator, in the mothur package [63]. 336 OTUs (12,308 clones) contributed to this analysis. Cluster dendrograms, with added bar charts showing the microbial composition of each sample and Shannon Diversity Indices, were visualized using the iTOL web package [65]. For the PCA plot OTUs were generated as above but with a 97% identity cut-off. PCA decomposition was performed on the (symmetric) matrix of pairwise sample similarity, where the similarity metric was based on the sum of absolute differences in OTU frequency. 344 OTUs (16,154 clones) contributed to the analysis, which was insensitive to the removal of low frequency OTUs.

To determine the SCFA profile, the cecal contents from 5 mice per group were pooled and then resuspended in sterile PBS at a concentration of 500 mg/ml, homogenized and centrifuged at 14,000 rpm for 10 minutes. Supernatant was collected, acidified and following conversion to *t*-butyldimethylsilyl derivatives were analyzed by gas chromatography [66].

### Whole genome sequencing and phylogenetic analysis of intestinal bacteria

We sequenced the genomes (and their closest equivalent human-derived species) using the MiSeq platform, and performed *de novo* assembly using Velvet {[67] and gene prediction using GLIMMER3 [68]. We then identified the genes that were ubiquitous between the 6 MixB species, and reference intestinal bacterial genomes sourced from the MetaHIT project, the HGMI project, and the Human Microbiome Project (Table S5). 44 Common genes were identified using TBLASTN [69] searches against the complete dataset of the reference and assembled genomes for 80 bacteria (Table S5). Although the “true” core genome amongst these samples may be higher – we were limited by the fact that in several cases only draft assemblies were available, and so some genes which may have been expected to be present in the “core” group, were in fact not present, due to their absence in one or more of the draft genome sequences used. A gene was classified as being ‘present’ if it had a minimum percent amino acid identity across the entire gene of 30% compared to the reference. The reference genes used for querying were taken from the strain of *Staphylococcus warneri* derived from MixB. The common genes so identified were manually checked, translated, extracted, and concatenated together. We then used FastTree 2.1 [70], with its default settings (BLOSUM45 and the Jones-Taylor-Thorton CAT model, with 20 rate categories), to generate a maximum likelihood phylogeny from the concatenated protein sequence, in order to place the bacteria into their correct context and to distinguish species.

## Supporting Information

Figure S1 **Toxin A production by** ***C. difficile*** **027/BI-7, 012/630 and 017/M68.** *C. difficile* 027/BI produced TcdA at 200.3 ng/µl, *C. difficile* 630/012 produced TcdA at 21.5 ng/µl and *C. difficile* M68/017 does not produce TcdA. Data are from 3 independent experiments with triplicate determinants in each.
(EPS)

Figure S2 ***C. difficile*** **supershedders are highly contagious.** Donor mice (from Figure 1) infected with the indicated *C. difficile* variant were housed for 1 hour in sterile cages without bedding and then feces was removed and cages were left overnight so that only spore contamination remained. The next day naïve recipient mice were aseptically placed in cages for 1 hour and then aseptically removed and housed individually in sterile cages and given clindamycin in their drinking water. After 4 days the recipient mice were sampled to determine if they were infected with *C. difficile*. The transmission efficiency represents the percentage of recipient mice that became infected with *C. difficile*. Experiments were repeated at least twice and included 10 recipient mice per experiment. n.d = not determined.
(EPS)

Figure S3 **Expression microarray using cecal tissue of C57BL/6 mice supershedding either** ***C. difficile*** **027/BI-7 or 017/M68 at 5 days post-infection.** Red indicates upregulation and green indicates downregulation of genes compared to naïve, clindamycin treated control mice. Summary of data in Table S1.
(EPS)

Figure S4 **Distinct intestinal microbiota community structures from healthy/naïve mice (n = 17), clindamycin supershedders (** ***C. difficile*** **027/BI-7 infected mice on clindamycin; n = 10) and persisting supershedders (** ***C. difficile*** **027/BI-7 infected mice not on clindamycin; n = 17).** a) Plot illustrating the percentage of *C. difficile* 16S rRNA gene clones in libraries of healthy/naïve mice (n = 4,926 clones), clindamycin supershedders (n = 4,433 clones) and persisting supershedders(n = 2,956 clones). b) Comparison of SDI for the intestinal microbiota of healthy/naïve mice, clindamycin supershedders and persisting supershedders. Wilcoxon rank sum test was used to compare differences between groups.
(EPS)

Figure S5 **Fecal bacteriotherapy suppresses** ***C. difficile*** **intestinal colonization and diversifies the intestinal bacterial community of supershedder mice.** a) High-level excretion of *C. difficile* is rapidly suppressed after oral inoculation of supershedder mice with homogenized feces from a healthy mouse (input feces). Plotted red line represents average shedding levels of 5 mice and error bars indicate standard deviation. Black vertical arrow indicates day 58 when healthy feces was administered and green arrowheads indicate the times when fecal DNA was extracted for 16S rRNA gene analysis. b) Composition of intestinal bacterial community of supershedder mice (n = 2) shifts to reflect that from the healthy donor mouse after bacteriotherapy. c) Diversity of intestinal microbiota of supershedder mice increases after bacteriotherapy as indicated by an increase in the Shannon Diversity Index scores.
(EPS)

Figure S6 **Impact of various oral treatments on epidemic** ***C. difficile*** **027/BI supershedder state in mice.** Fecal shedding profile from supershedder mice (n = 5/group) that were treated with feces or fecal derivatives. Standard treatments with a) feces and b) PBS are the same as in Figure 2. The following treatments were administered into supershedder mice via oral gavage with a 200 µl volume. c) Equivalent feces was autoclaved using standard conditions and then resuspended in sterile PBS for a final concentration of 100 mg/ml. d) To produce fecal filtrate, feces was homogenized in sterile PBS at a concentration of 100 mg/ml and then centrifuged at 14,000 rpm for 10 minutes to separate the bacteria/particulate matter from the soluble fraction which was then filtered through a 0.22 µm filter. This was referred to as the fecal filtrate. e) SCFA indicates a mixture of acetate∶propionate∶butyrate in a ratio of 6∶1∶2 at a concentration of 100 mM that was at pH 6.5. f) Lab adapted *E. coli* strain C600 (nalidixic acid resistant) was gavaged into mice at a dose of 10^8^ CFU. *E. coli* colonization was confirmed by culturing feces of supershedder mice. The broken horizontal line indicates the detection limit.
(EPS)

Figure S7 **Rarefaction curves demonstrating observed bacterial diversity of feces from healthy, naïve mice and its serially passaged derivatives.** In addition, the Chao1 calculator estimated the total community diversity (OTU defined at ≥98% similarity) for the healthy feces as 142 phylotypes (95% confidence interval 105–225), passage 1 as 30 phylotypes (95% confidence interval 27–46), passage 2 as 6 phylotypes (95% confidence interval 5–18) and passage 3 as 4 phylotypes (95% confidence interval 4-4). Together, these results demonstrate that serial passage of healthy feces in nutrient broth progressively reduced the complexity of the bacterial community.
(EPS)

Figure S8 **Simplified fecal derivatives enriched for easily culturable components effectively suppress the epidemic** ***C. difficile*** **supershedder 027/BI state in mice.** a) Fecal shedding profiles from supershedder mice (n = 5/group) that were treated with healthy feces, a *Bacteroides/Lactobacillus* mixture (*Bacteroides acidifaciens*, *Bacteroides vulgatus*, *Lactobacillus murinus* and *Lactobacillus reuteri*), feces cultured in Wilkins-Chalgren Anaerobic broth at 37°C either aerobically or anaerobically. Pie charts illustrate the composition of the input treatments based on 16S rRNA gene clone libraries for healthy feces, aerobic passaged and anaerobic passaged inputs or based on culturing for the *Bacteroides/Lactobacillus* mixture. b) Shannon Diversity Indices of the intestinal microbiota of supershedders pre- and post-treatment (day 3, 4, 6 and 14) and that of the corresponding input community.
(EPS)

Table S1 **Differential gene expression between** ***C. difficile*** **027/BI7 and 017/M68 infected cecal tissues.** Host gene expression was assessed on mice infected with either *C. difficile* 027/BI7 or *C. difficile* 017/M68 for 5 days or clindamycin-treated uninfected controls. Genes differentially expressed between the two infected groups are shown in the table.
(XLSX)

Table S2 **Summary of 16S rRNA gene clone library data used in this study.** 19,991 sequences, generated from a total of 87 samples, were included in the study.
(XLS)

Table S3 **Bacterial species isolated from cultured fecal derivative.**
(DOC)

Table S4 **Summary of data used whole genome phylogeny of intestinal bacteria presented in** **Figure 5** **.**
(XLSX)

Table S5 **Primers used for RT-PCR experiments shown in** **Figure 3** **.**
(DOCX)
