# Supplementary material for: Targeted Restoration of the Intestinal Microbiota with a Simple, Defined Bacteriotherapy Resolves Relapsing Clostridium difficile Disease in Mice
Source: PLoS Pathog. 2012 Oct 25;8(10):e1002995. doi: 10.1371/journal.ppat.1002995 (PMC3486913; doi:10.1371/journal.ppat.1002995)
Supplement: Table S3 — Bacterial species isolated from cultured fecal derivative. (DOC) [file ppat.1002995.s011.doc]

**Supplemental Table 3**. Bacteria isolated from cultured fecal derivative. Species designation is based on the sequence of the 16S rRNA gene or whole genome sequencing and comparative genomics using the genomes of intestinal bacteria.

| Mix | Species based on 16S rRNA gene | Genus Species based on WGS | Phylum |
| --- | --- | --- | --- |
| A | *Bacteroides acidifaciens* |  | *Bacteroidetes* |
| A | 16saw22-1a06.p1k, *Barnesiella intestinihominis* (87%) |  | *Bacteroidetes* |
| A | *Lactobacillus taiwanensis/gasseri/johnsonii* |  | *Firmicutes* |
| A | *Flavonifractor plautii* |  | *Firmicutes* |
| A | R-7912, *Turicibacter sanguinis* (97%) |  | *Firmicutes* |
| A | *Bifidobacterium pseudolongum* subsp. *globosum/pseudolongum* |  | *Actinobacteria* |
| A | *Escherichia coli* |  | *Proteobacteria* |
| B | 16saw22-1a06.p1k, *Barnesiella intestinihominis* (87%) | *Bacteroidetes* novel species | *Bacteroidetes* |
| B | *Lactobacillus reuteri* | *Lactobacillus reuteri* | *Firmicutes* |
| B | *Enterococcus hirae/faecium/durans* | *Enterococcus hirae* | *Firmicutes* |
| B | *Anaerostipes caccae/Clostridium indolis* | *Anaerostipes* novel species | *Firmicutes* |
| B | *Staphylococcus warneri/pasteuri* | *Staphylococcus warneri* | *Firmicutes* |
| B | WD3_aako2b03, *Adlercreutzia equolifaciens* (97%) | *Enterorhabdus* novel species | *Actinobacteria* |
| C | *Parabacteroides distasonis* |  | *Bacteroidetes* |
| C | 16saw22-1a06.p1k, *Barnesiella intestinihominis* (87%) |  | *Bacteroidetes* |
| C | *Lactobacillus murinus/animalis* |  | *Firmicutes* |
| C | *Enterococcus faecalis* |  | *Firmicutes* |
| C | *Blautia producta* |  | *Firmicutes* |
| C | *Propionibacterium acnes* |  | *Actinobacteria* |
| C | *Acinetobacter lwoffii/baumannii* |  | *Proteobacteria* |
| B1 | WD3_aako2b03, *Adlercreutzia equolifaciens* (97%) | *Enterorhabdus* novel species | *Actinobacteria* |
| B1 | *Anaerostipes caccae/Clostridium indolis* | *Anaerostipes* novel species | *Firmicutes* |
| B1 | *Staphylococcus warneri/pasteuri* | *Staphylococcus warneri* | *Firmicutes* |
| B2 | 16saw22-1a06.p1k, *Barnesiella intestinihominis* (87%) | *Bacteroidetes* novel species | *Bacteroidetes* |
| B2 | *Lactobacillus reuteri* | *Lactobacillus reuteri* | *Firmicutes* |
| B2 | *Enterococcus hirae/faecium/durans* | *Enterococcus hirae* | *Firmicutes* |
