# Supplementary material for: Targeted Restoration of the Intestinal Microbiota with a Simple, Defined Bacteriotherapy Resolves Relapsing Clostridium difficile Disease in Mice
Source: PLoS Pathog. 2012 Oct 25;8(10):e1002995. doi: 10.1371/journal.ppat.1002995 (PMC3486913; doi:10.1371/journal.ppat.1002995)
Supplement: Table S5 — Primers used for RT-PCR experiments shown in Figure 3 . (DOCX) [file ppat.1002995.s013.docx]

**Supplementary Table 5:** Sequences of primers and probes used for quantitative RT-PCR.

| **Primer name** | **Sequence** |
| --- | --- |
| Gapdh F | 5’-TGTGTCCGTCGTGGATCTGA-3’ |
| Gapdh R | 5’-CACCACCTTCTTGATGTCATCATAC-3’ |
| Gapdh probe* | 5’-TGCCGCCTGGAGAAACCTGCC-3’ |
| IL-6 F | 5’-ACAAGTCGGAGGCTTAATTACACAT-3’ |
| IL-6 R | 5’-TTGCCATTGCACAACTCTTTTC-3’ |
| IL-6 probe* | 5’-TTCTCTGGGAAATCGTGGAAATG-3’ |
| iNOS F | 5’-TGCATCGGCAGGATCCA-3’ |
| iNOS R | 5’-AACATTTCCTGTGCTGTGCTACA-3’ |
| iNOS probe* | 5’-CCTGCAGGTCTTTGACGCTCGGAA-3’ |
| Ly6G F | 5’-TGCCCCTTCTCTGATGGATT-3’ |
| Ly6G R | 5’-TGCTCTTGACTTTGCTTCTGTGA-3’ |
| Ly6G probe* | 5’- TGCGTTGCTCTGGAGATAGAAGTTATTGTGGACT-3’ |

*Probes were labeled with FAM (5’) and TAMRA (3’).
